# Smart Lids for deep multi-animal phenotyping in standard home cages

**DOI:** 10.3389/fnbeh.2025.1696654

**Published:** 2026-01-20

**Authors:** Sead Delalić, Michael Kaca, Pratomo Alimsijah, Noah Weber, Elmedin Selmanović, Mikailynn Galindez, Glen Marquez, Francisco Balmaceda, Eldina Delalić, Iman Bekkaye, Lejla Bakija, Meliha Kurtagić-Pašalić, Esma Agić, David Anderson, Amy Wagers, Michael Florea

**Affiliations:** 1 Olden Labs PBC, South San Francisco, CA, United States; 2 Next IT d.o.o, Sarajevo, Bosnia and Herzegovina; 3 Department of Stem Cell and Regenerative Biology, Harvard University, Cambridge, MA, United States

**Keywords:** 3R, AI, animal welfare, computer vision, home-cage monitoring, multi-animal tracking, phenomics, phenotyping

## Abstract

The reproducibility crisis and translational gap in preclinical research underscore the need for more accurate and reliable methods of health monitoring in animal models. Manual testing is labor-intensive, low-throughput, prone to human bias, and often stressful for animals. Although many smart cages have been introduced, they have seen limited adoption due to either low throughput (being limited to single animals), low data density (a few metrics only), high costs, a need for new space or infrastructure in the vivarium, high complexity use, or a combination of the above. Although technologies for video-based single-animal tracking have matured, no existing technology enables robust and accurate multi-animal tracking in standard home cages. To solve these problems, we built a new type of assay device: the Smart Lid. Smart Lids mount to existing racks, above standard home cages and stream video and audio data, turning regular racks into high-throughput monitoring platforms. To solve the multi-animal tracking problem, we developed a new computer vision pipeline (MOT - Multi-Organism Tracker) along with a new ear tag purpose-designed for computer vision tracking. MOT achieves over 97% accuracy in multi-animal tracking while maintaining an affordable runtime cost (less than $100 per month). The pipeline returns 21 health-related metrics, covering activity, feeding, drinking, rearing, climbing, fighting, cage positioning, social interactions and sleeping, with additional metrics under development.

## Introduction

Animal models are indispensable in biomedical research and drug development. However, they face well-documented challenges in translatability and data reliability (Mukherjee et al., 2022; Wendler and Wehling, 2010). A significant majority of the findings of preclinical animal studies do not translate into effective human treatments. For example, almost 90% of the drug candidates that pass animal testing ultimately fail in human trials (Sun et al., 2022). Approximately half of these failures are attributed to unanticipated toxicities in humans that were not detected in animal studies (Sun et al., 2022). Such stark discrepancies highlight poor translational accuracy and raise concerns that current animal testing paradigms often do not accurately predict human outcomes. Another major challenge in animal studies is that important phenotypic changes may be missed or detected only after significant delays when using conventional methods. These factors contribute to a “Valley of Death” between animal research and clinical application, where promising preclinical results often fail to yield human benefits. Yet, animals continue to be vital for many aspects of biomedical research. Consequently, there is a pressing need for methods that enhance data throughput, objectivity, and relevance *in vivo*.

Home-cage monitoring (HCM) has emerged as a promising approach to address these issues (Mingrone et al., 2020; Richter, 2020). In HCM, animals are continuously observed in their familiar home-cage environment, without the stress of handling or novel test arenas (Gouveia and Hurst, 2013). This approach offers several advantages: (a) data can be collected 24/7, capturing complete activity cycles and rare events; (b) the scored behaviors are spontaneous and produced by the animals in their living environment, increasing the ethological validity of the behavioral testing, (c) animals are undisturbed, reducing stress (Balcombe et al., 2004); (d) the method aligns with the ethical principles of the 3Rs (Replacement, Reduction, Refinement) by refining how experiments are conducted and by potentially reducing the number of animals needed.

Despite its recognized utility, broad adoption of HCM has been limited. Implementing an effective monitoring system is inherently complex, requiring integration of specialized hardware, continuous data acquisition, large-scale data management, and sophisticated behavior analysis algorithms (Fuochi et al., 2024). Early automated systems often required significant technical expertise to set up and operate and were typically limited to measuring simple metrics (e.g. overall activity or location) rather than a wide repertoire of behaviors. This complexity barrier has slowed the spread of HCM beyond specialized laboratories. Furthermore, many commercially available systems cost $10,000 or more per cage and/or are limited to measuring a single animal at a time, leading to major accessibility and throughput barriers. Single housing additionally imposes limitations on the types of studies and the length of studies that can be conducted, since animals require social contact for long-term well-being. As a result, by and large, manual assays have remained the most cost-effective and flexible method for most laboratories.

Recent advances in computer vision and machine learning are helping overcome previous limitations in home-cage monitoring. AI-driven platforms now enable accurate video tracking and behavior recognition, allowing fully automated analysis (Mathis et al., 2018). Open-source tools have shown that short-term multi-animal tracking is feasible in test setups in lab animals (Pereira et al., 2022; Romero-Ferrero et al., 2019; Chen et al., 2023; Segalin et al., 2021), and integrated systems combining hardware and AI have emerged. Notable examples include the DVC^®^ (Tir et al., 2025) and UID Mouse Matrix systems, using capacitance and RFID respectively, and video-based platforms like DIV Sys for single-animal analysis (Robertson et al., 2024). Building on these developments, we sought to develop a system that enables long-term multi-animal tracking from the home cage, at a cost that is lower than the cost of manual analysis.

In this paper, we describe the development of the Smart Lid^TM^ devices that replace or fit above existing cage lids and record video, audio and other types of data, enabling use of existing IVC cages and racks. Specifically, we developed a cloud-connected Digital Online Monitoring Equipment (DOME) Smart Lid^TM^ version along with a dedicated cloud-based computer-vision pipeline for multi-animal tracking. We validate the system's accuracy against human annotations and assess the effects of high fat diet, ethanol consumption and aging on a panel of health metrics.

## Methods

Our goal is to detect, identify, and track multiple mice over extended periods, and to infer relevant behavioral patterns with minimal human intervention. To do so, the pipeline integrates video acquisition, object detection, identity tracking, occlusion handling, and post-processing for behavior quantification. Each component is designed to be robust to variations in lighting, animal posture, and cage enrichment configurations, enabling scalable and reliable analysis across diverse experimental setups.

### Video acquisition and pre-processing

All data presented in this study were collected using Allentown Jag75 cages and the metrics reported here apply specifically to the Allentown configuration. DOME Smart Lids^TM^ perform continuous (“always-on”) recording and maintain 7 days of local storage on the onboard computer. In the event of network interruption, the locally stored video is used to automatically backfill to the cloud once connectivity is restored, preventing data loss. All devices use Network Time Protocol (NTP) for clock synchronization, with periodic drift correction to maintain timestamp accuracy over multi-day recordings. Video is compressed using a proprietary codec and bitrate configuration that preserve frame timing and analysis fidelity. All behavioral recordings were captured using overhead cameras at a resolution of 640 × 480 pixels in grayscale format. Videos were acquired and stored at 10 frames per second to ensure computational efficiency and reduced storage requirements without compromising behavioral resolution. This format and framerate are consistently applied across all cage types. The camera field of view is fixed to cover the entire cage area. DOME^TM^ devices do not alter the environmental conditions of the cage: air quality and temperature measurements indicate equivalence between cages equipped with regular lids and with DOMEs (Supplementary Figure 1).

### Mouse identification and tracking

Mouse identity is maintained across frames using a tracking-by-detection approach (Redmon et al., 2016; Bochkovskiy et al., 2020). To associate detections over time, the system employs a variation of optical flow (Lucas and Kanade, 1981) tailored to the constrained movement patterns observed in cage environments. At the start of each session, unique identifiers are assigned to individual mice, either manually or automatically based on detected ear tags. When detections are temporarily missing (due to occlusions or frame-level noise) the system predicts positions and propagates identities forward using prior spatial and motion estimates. To preserve identity consistency over long durations, additional heuristics are applied to manage potential identity switches. These include spatial consistency checks, motion continuity constraints, and tag visibility cues. Combined, these strategies ensure robust long-term tracking with no observed drift or degradation in identity assignment, even during extended monitoring periods.

### Switching logic for occlusions and identity maintenance

During occlusions and interactions, bundle detections trigger specialized switching logic. The system uses motion extrapolation, spatial context, and custom animal heuristics to resolve ambiguous identity assignments. Confidence multipliers are applied based on consistency with predicted trajectories and model detection scores. Identity switches are deferred unless supported by multiple instances of high-confidence evidence. This conservative strategy reduces false-positive ID swaps and ensures continuity through high-contact interactions.

### Post-processing and behavior analysis

Trajectories are temporally smoothed, and activity metrics including acceleration, angular motion, average movement speed, max movement speed and distance traveled are computed from animal centroid coordinates. Aggression and social distance is analyzed through calculating the distances as well as movement trajectories of animals, as described in the section *Health and Behavior Metrics*. Cage exchange events are derived using historical frame data. Time spent in various locations of the cage is calculated from the body centroid coordinates. Time spent climbing, rearing, eating, drinking and interacting with enrichment is calculated from a combination of body and head coordinates, direction and regions of interest of target objects. Sleep bouts and sleep time is estimated from body and head coordinates. Detailed descriptions of each metric is listed in the section *Health and Behavior Metrics*. The full pipeline is optimized for multi-day behavioral monitoring with minimal drift, enabling fully automated experiments.

### Health and behavior metrics

Health and behavior metrics were calculated as described below. Metrics are calculated from a combination of body or head centroid location, head direction, movement velocity and direction and/or overlap of bounding boxes of animals to animals or animals to regions of interest.

#### Activity and movement

Acceleration (cm/s^2^): Average linear acceleration while the animal is moving (excludes quiescent/sleep periods).

Angular motion (deg/s): Average movement angular velocity while moving.

Average movement speed (cm/s): Mean linear speed over the selected observation period (time bin), including inactivity.

Average awake movement speed (cm/s): Mean linear speed over the selected observation period (time bin), excluding at periods of inactivity.

Max movement speed (cm/s): Mean of the top 1% of frame-wise movement speeds during active periods (reduces noise from single-frame spikes).

Distance traveled (cm): Total travel path length over the observation period.

#### Social behavior

Aggression events (count): Count of individual aggression bouts involving chasing, mounting, fighting or combination of the three and involving two or more animals during the selected observation period (time bin). From our observation, animals tend to engage in aggression bouts lasting from 20 s to 5 min where, with the exception of a few second pause, they engage in one of the three behaviors above. We count all aggressive behaviors in such bout as one overall aggression “event”.

Aggression time (%): Percentage of time the animal is engaged in aggression (chasing, mounting, fighting, or a combination of the three).

Social distance (cm): Average distance of the mouse's centroid to the nearest mouse centroid, averaged over the observation period (time bin). Note that the metric does not consider directionality of animals - therefore a low social distance does not necessarily imply interactions. The main purpose of this metric is to identify social distancing or withdrawal.

#### Spatial occupancy

Cage center time (%): Percentage of time in the center zone (≥2 cm from all cage walls).

Cage front time (%): Percentage of time in the front half of the cage.

Cage corner time (%): Percentage of time spent in the approximately 9 cm^2^ area of the 4 cage corners.

Enrichment tube interaction time (%): Time spent on, in, or immediately around the enrichment tube (e.g., standing on top, inside, sitting, or rearing against), determined by position tracking.

#### Climbing and rearing

Climb time (%): Percentage of recording time spent climbing on the food hopper metal grid, defined as both feet off the floor and reaching the upper portion of the hopper.

Climbing events (count): Count of discrete climbing bouts as defined above.

Rearing events (count): Count of rears: animal standing with two feet against one of the three side walls, excludes rears against the food hopper, red tube or in the cage center.

Time spent rearing (%): Percentage of recording time spent rearing as defined above, excludes rearing against the food hopper, red tube or in the cage center.

Center rears and hopper rears were not separately annotated or detected. Accurate identification of these subtypes would require additional pose-estimation models to determine the relative position of the animal to cage structures. Because rears in our datasets occurred predominantly at walls or near enrichment and were similarly distributed across treatment groups, restricting analysis to wall rears is unlikely to introduce systematic bias.

#### Feeding and drinking

Drinking time (%): Time spent interacting with the bottle nozzle or autowater spout.

Eating time (%): Time spent interacting with food at the hopper, includes “grinding” (chewing without ingestion).

Feeding and drinking metrics quantify interaction time with the food hopper or water spout. These measures reflect behavioral engagement rather than actual intake, which can vary substantially depending on the age, behavior and dental health of animals.

#### Sleep and inactivity

Sleep bouts (count): Number of discrete inactivity bouts lasting ≥2 minutes. The 2 min threshold was selected based on Pack et al. (2007) that demonstrated that the majority of sleep events can be captured with a 2 minute inactivity time or longer.

Sleep time (%): Percentage of recording time classified as sleep (continuous inactivity ≥2 minutes).

#### Digital Bioage

The Digital Bioage is a composite metric that aims to track the chronological age of mice. It was generated by assessing 8 behavioral parameters (Time spent rearing and Rearing events, Average and Max movement speed, Movement acceleration, Climbing events, Distance traveled and Time spent sleeping) in a total of N=19 male and female C57BL/6J mice ranging from 113 days old to 933 days old (age and sex details in Supplementary Table 1). Each cohort of mice was measured for at least 48 hours. Linear regression was used to generate a set of weights for each metric to provide one compound number that correlates with the chronological age of mice.

### Animal experiments

All animal experiments were carried out in a facility overseen by a veterinarian and certified by the state of California to house research mice. All experimental procedures involving animals were conducted in accordance with institutional, national, and international guidelines for the care and use of laboratory animals. The study protocol was reviewed and approved by an internal ethics board prior to the start of the study. Animal welfare was given the highest priority throughout the study and all efforts were made to minimize suffering and distress. Olden Labs is approved to house research animals by the State of California Department of Public Health (license #113).

The animals were used strictly for scientific purposes, as described in the approved protocol. The number of animals was kept to the minimum required to achieve statistically significant results, following the principles of Replacement, Reduction, and Refinement (the 3Rs). Humane endpoints were predefined (sudden loss of weight of more than 20% of body mass, torpor, reduction of body temperature below 35 degrees Celsius, non-reaction to physical stimulus, open lesions larger than 4mm in diameter), and animals were monitored regularly for clinical signs of distress or pain. Humane endpoints were not met during the course of the studies for any animals.

Animals (C57BL/6J or BALB/c mice) were purchased from Jackson Labs and acclimated to the local facility since 3 weeks of age. For Figure 4D, randomization was performed at the animal level to assign individual mice within mixed cages to ethanol vs. vehicle treatment. For Figures 5A, B, randomization was performed at the cage level using an online randomizer (random.org). For Figure 5C, Supplementary Figure 5, all available aged animals were included; these fixed cohorts precluded randomization.

Animals were housed under standard laboratory conditions, including controlled temperature, humidity, and a 12-hour regular light/dark cycle (light period from 8 AM to 8 PM local time). Food (Kaytee Forti-Diet Pro Health) and water were available *ad libitum* unless otherwise noted. For the high-fat diet (HFD) experiment (Figure 5A), mice were fed Research Diets D12451 (Research Diets Inc., New Brunswick, NJ, 45% kcal from fat, 35% carbohydrate, 20% protein). All cages received diet from the same lot. Body weight was measured once at the start of the study, reported in Supplementary Figure 3. Eating time in our MOT pipeline quantifies interaction with the food hopper (retrieval, chewing, grinding) rather than total caloric intake (hopper mass change was not recorded in this experiment). Environmental enrichment in the form of red tubes and/or wood sticks was provided to promote natural behaviors and well-being. Health status was monitored daily by trained personnel.

For the ethanol experiments (Figures 4D, 5B), 3-month-old female C57BL/6J mice were used. Estrous cycle stage was not monitored, which may contribute to within-group variation in activity and social measures. All animals were age-matched and acclimated under identical housing and environmental conditions.

Animals were ear tagged unanesthetized using a modified three-finger scruff method according to the guidelines described in Supplementary File 1—Ear Tagging Guide. Animals were acclimatized to ear tags for at least 4 days prior to experimentation. Animals received from external vendors were acclimatized for at least 14 days prior to experimentation.

### Statistical analysis

For each experiment, randomization and blinding procedures were implemented as follows. For the high-fat diet (HFD) study (Figure 5A), animals were randomized at the cage level and assigned to regular diet control group or HFD group using an online randomizer (random.org). For the ethanol study (Figures 4D, 5B), animals were randomly assigned to treatment vs. vehicle at the animal level for mixed-treatment cages (Figure 4D), and at the cage level for the full-cage dosing experiment (Figure 5B), again using random.org. For the aging study (Figure 5C), all available aged animals were included, therefore, randomization was not applicable.

All behavioral metrics are binned over 24h windows unless otherwise stated. Where indicated, light-phase and dark-phase values are computed together. Summary values reflect the average across the total time recorded.

Manual annotation of videos for validation of MOT accuracy was performed by annotators who were fully blinded to treatment group and experimental condition. Automated behavioral analysis was performed by the MOT pipeline, which does not access treatment metadata during inference, ensuring unbiased extraction of behavioral metrics. Experimenters performing injections or cage assignment were blinded to group identity until analysis was complete.

Power analysis was performed *post hoc* for the HFD and ethanol studies based on pilot variance estimates and expected effect sizes reported in prior literature for locomotor and rearing-related outcomes. Group sizes were selected to provide ≥80% power to detect moderate to large behavioral effects. For the aging cohort, power calculations were not applicable due to use of fixed-availability age groups; variance and sample-size constraints are detailed in the Supplementary material.

Statistical significance was assessed via multiple unpaired **t**-tests using False Discovery Rate (FDR) approach to correct for multiple comparisons at FDR Q = 5%. All statistical analysis was performed using Graphpad Prism 9. Details of tests are described in figure legends.

### Data availability

Representative raw video excerpts, derived behavioral summaries, and the numerical data underlying key figures are available at https://drive.google.com/drive/folders/1ZJBuFehQpre0XMz3r7P8LIu4VxLoQVFO. These materials are sufficient to illustrate the processing steps and reproduce the aggregate analyses shown in the manuscript.

The full MOT pipeline, including trained model weights, proprietary feature-extraction code, internal annotation schema, and the complete labeling interface, forms part of a commercial software stack and cannot be publicly released. High-level descriptions of the annotation workflow and the model-versioning scheme are provided in the Supplementary material. Additional de-identified example data may be provided upon reasonable request.

## Results

### Smart Lids enable data collection in existing racks

**Figure 1:**
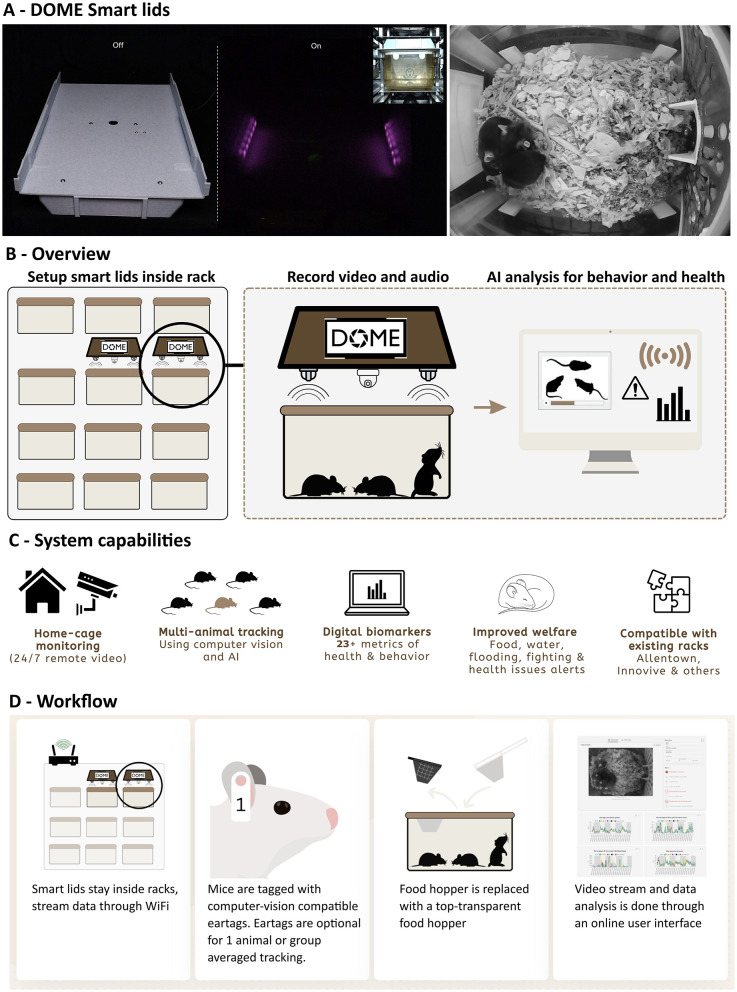
Smart Lids. **(A, B)** DOME Smart Lids fit above mouse home cages in existing racks. They record and stream video and audio data, which is then analyzed for keypoint detection and subsequent estimation of activity, behavior and health parameters by a computer vision pipeline (MOT). Smart Lids illuminate the cage with near infrared light and record video at the near infrared spectrum, resulting in monochromatic and consistent video feed across the day/night cycle. **(C)** DOME Smart Lids are cloud-connected and enable 24/7 real-time home-cage monitoring, multi-animal tracking, subsecond-resolution estimation of behavior and health, email alerts on cage conditions and health, and are compatible with a variety of existing home-cage racks. The data presented in this paper is gathered from the Allentown Jag75-compatible system. **(D)** The system is setup by placing DOME Smart Lids inside racks (inset) and connecting them to local WiFi/Ethernet. Smart Lids stay within the rack and do not move with the cage. If multi-animal tracking is required, mice are then tagged with ear tags purpose-designed for computer vision tracking. The existing food hopper is replaced by a custom food hopper that is transparent from the top, and video stream and data are accessible through the user interface at oldenlabs.com.

**Figure 2:**
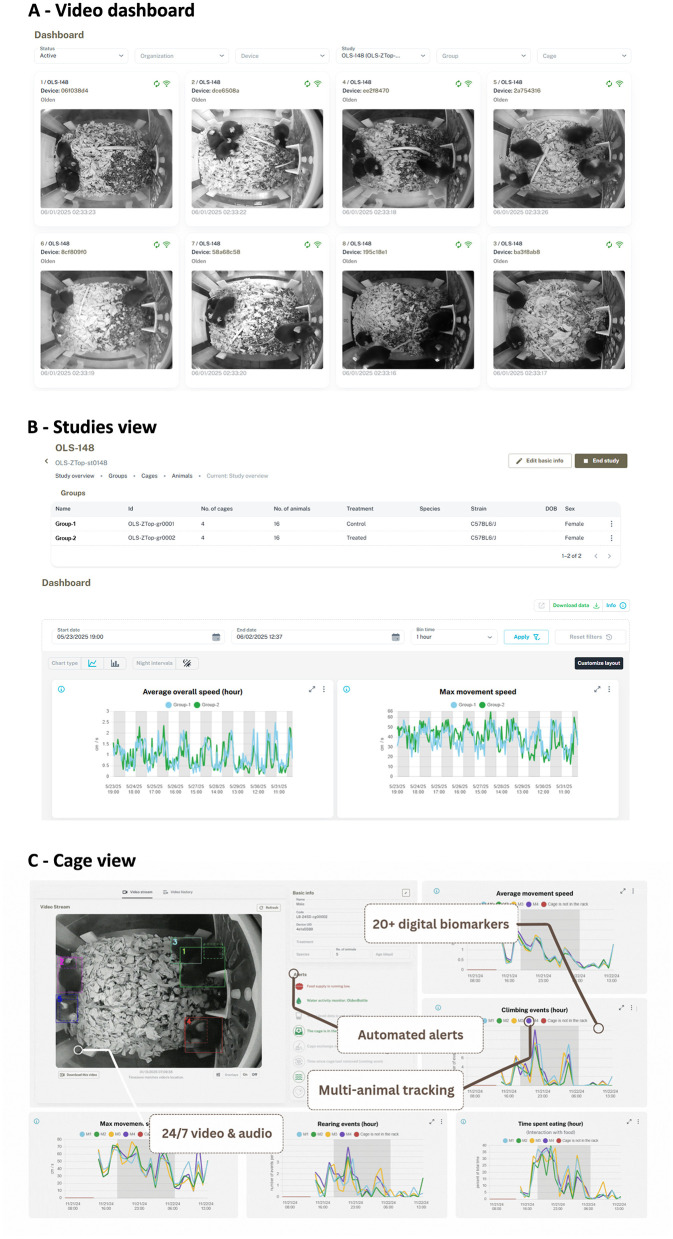
User interface. The interface is divided into three main parts. **(A)** Video overview, showing live feeds from all cages. **(B)** Studies view for studies setup, data analysis and export between groups. **(C)** Cage view, showing individual animal metrics, cage condition alerts and metrics overlays.

**Figure 3:**
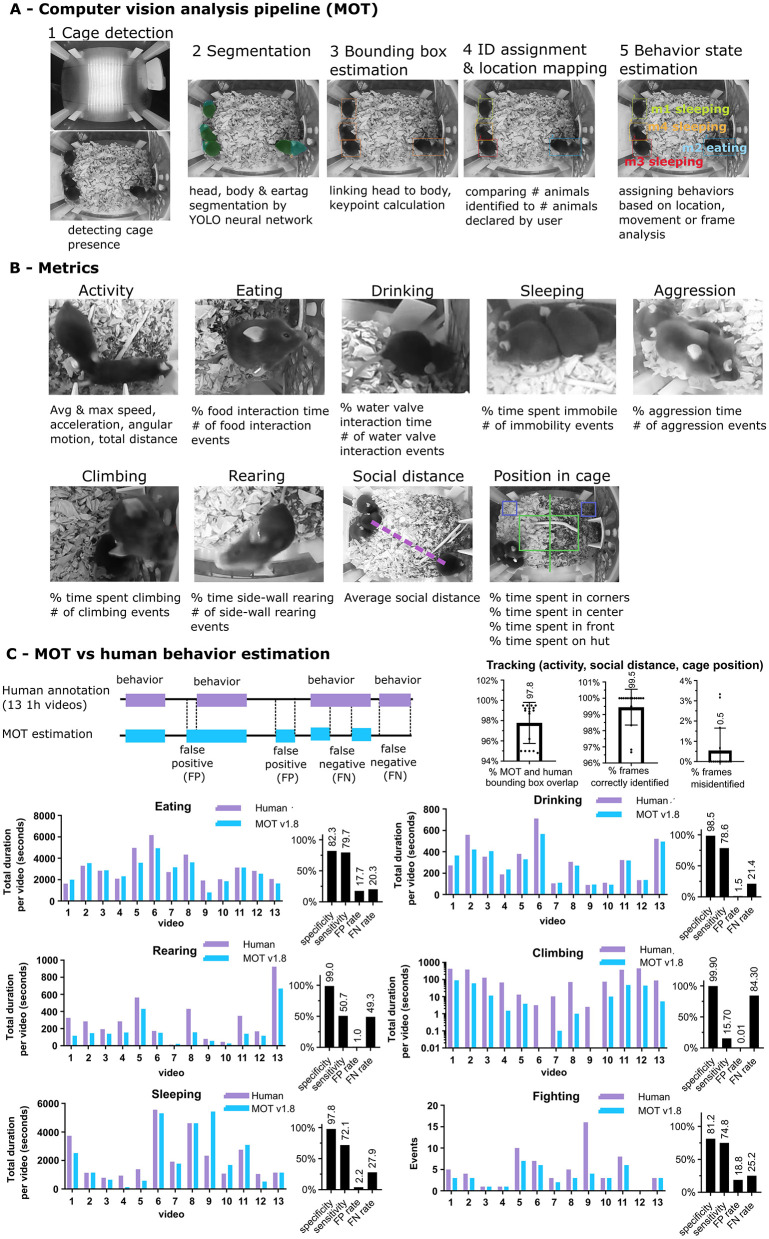
Computer vision pipeline. **(A)** Video analysis pipeline (MOT—Multi-Organism Tracker). First, MOT pipeline looks for presence of the cage. If found, a YOLO-esque neural network is called to segment the body, head and ear tag (if present). Heads are linked to bodies based on distance and other heuristics. The head and body bounding boxes and keypoints are calculated from the segmentation masks. ID is then assigned to each animal based on ear tags. For untagged animals, IDs are assigned randomly. Locations of bounding boxes and head and body centroids are logged for each frame. Then, activity, behavior and location metrics are calculated. **(B)** In total 21 metrics are calculated across 9 categories based on location, direction, motion or raw frame analysis. **(C)** Comparison of event annotation between the latest MOT (v1.8) and human annotation. 13 one-hour videos from cages containing 2–5 black (C57BL/6J) or white (BALB/c) male and female mice (total:23; black:18; white:5) were timestamp annotated by human annotators and cumulative time of each behavior from all mice was recorded. First, bounding boxes drawn by human annotators were compared to MOT-generated bounding boxes to assess the overall accuracy of mouse identification, showing more than 97% overlap between human and MOT bounding boxes and correct identification rate of 99.5% frames. Annotated behaviors were then compared against MOT predicted events. For each metric, the false-positive rate (where MOT identifies an event where there is none) and false-negative rate (MOT fails to identify an event) were calculated. Specificity is 1-FP rate and sensitivity 1-FN rate. Note that MOT was optimized for high specificity over high sensitivity.

Space is often limited in vivariums. Additionally, existing workflows built around cleaning and sterilizing existing cage bottoms impose substantial barriers to integrating any monitoring system that is not compatible with those workflows. For this reason, we sought to develop a system that integrated with existing individually ventilated cage (IVC) racks and existing cage bottoms. To do this, we developed devices that either replace the lid (for non-transparent lids) or fit above the existing cage lids (for transparent lids). For operational reasons, we started with the former option, and integrated with the Allentown Jag75 rack and cage bottom. To enable monitoring within this system, we developed a DOME Smart Lid that replaces the standard lid and is equipped with near infrared LEDs, near infrared fisheye camera, microphone, and WiFi-connected computer (Figures 1A, B). To enable use of existing cages, we also designed new food hoppers transparent to the camera at the top. The system enables 24/7 monitoring and analysis of behavior and health in multiple co-housed mice (Figure 1C). Use of the Smart Lids entails sliding the devices into the rack, connecting them to power, replacing the food hoppers and optionally tagging mice with ear tags. Once powered on, the devices automatically connect to the local WiFi, stream data, and store up to 7 days worth of data locally to buffer against loss of connectivity (Figure 1D). The system is easy to setup, requiring approximately 20 min for a person unfamiliar with the system, stays in the rack (i.e. does not need to be moved along with the cage), produces no visible light or sound, has no ports or water-sensitive areas, does not interfere with IVC airflow and does not come into direct contact with animals or animal exhaust air. Therefore, after installation, Smart Lids can simply remain in the rack and require little cleaning, meaning very low operational complexity or overhead for the user. As soon as the device is powered on, it starts recording and attempts to connect to a local internet (either through WiFi or Ethernet), and will continuously record regardless of whether a study has been initiated, as a failsafe against accidental loss of data. Defining a study in the user interface then triggers the analysis of the raw video and/or audio data for multi-animal tracking and health estimation. The data flows through a cloud provider and is accessible from an online user interface, removing the need for cumbersome local storage and data transfer. The user interface performs three main functions: (1) it enables access to a real-time video feed in a security-cam-like dashboard (Figure 2A); (2) it enables study setup and study data review (Figure 2B); (3) it enables review of individual animal data and cage conditions (Figure 2C). Video monitoring also makes it possible to effectively estimate cage conditions - therefore, we implemented automated alerts for low food, low mobility, flooding, and excessive fighting, with alerts for past due cage exchange or open skin lesions on the roadmap. Alerts are sent to users via email. The analyzed data is accessible from a dashboard on the interface, and enables visualization of AI tracking and download of analyzed data, raw video data, or pre-processed tracking data.

### Smart Lids enable accurate estimation of 21 health metrics

To analyze the resulting data, we developed a computer vision pipeline integrating neural networks and classical computer vision, specially designed to function with multiple animals in the context of the home cage, at low runtime cost (Figure 3A). First, the pipeline (MOT—Multi-Organism Tracker) analyzes the image to determine whether a cage is present. If so, the detection pipeline employs neural networks (Redmon et al., 2016; Bochkovskiy et al., 2020) to identify key components within the cage environment, including mouse bodies, heads, tags, food hopper, and other enrichment elements. The system is robust to variations in cage layout, lighting conditions, and mouse appearance, supporting both white and black mice of different sizes. Specialized models of varying complexity are used for distinct tasks, ranging from multi-object detection to classification. All models were trained on annotated video frames collected from home cages across diverse experimental conditions. To improve precision and efficiency, spatial heuristics are applied to handle edge cases such as reflections and partial occlusions. As an output, bounding boxes for head and body are drawn and linked. Each bounding box receives an ID, either from the detection of an ear tag (if present) or a randomly assigned ID if an ear tag is absent. The number and ID of identified animals are then compared to the user declared number and ID of animals, which is treated as ground truth. If no animals or wrong IDs are identified, an alert is sent to the user to correct the mistake. Treating user defined values as ground truth enables robust tracking of animals even during times of heavy occlusion or loss from vision (due to burrowing or enrichment). Finally, the resulting bounding boxes are propagated through a modified optical flow approach (Lucas and Kanade, 1981) or further calls of YOLO-esque segmentation networks (Redmon et al., 2016; Bochkovskiy et al., 2020). Bounding box coordinates, head centroids, body centroids, and head direction are recorded from each frame. Finally, behavior and health metrics are calculated from the combination of location, direction, velocity, segmentation mask or raw frame input analysis. The result is a file containing the location and behavior state of each animal in the cage for each frame.

From this data, 21 health metrics are estimated, encompassing activity, sleep, food intake, water intake, aggression, climbing, rearing, social distance and positioning in the cage (Figure 3B). The metrics are:

- Activity & Movement: Acceleration (cm/s^2^), Angular motion (deg/s), Average movement speed (cm/s), Average awake movement speed (cm/s), Max movement speed (cm/s), and Distance traveled (cm).
- Sleep : Sleep bouts (count) and Sleep time (%).
- Feeding and drinking: Drinking time (%) and Eating time (%).
- Climbing and rearing: Climb time (%), Climbing events (count), Rearing events (count), and Time spent rearing (%).
- Social behavior: Aggression events (count), Aggression time (%), and Social distance (cm).
- Spatial occupancy: Cage center time (%), Cage front time (%), Cage corner time (%), and Enrichment tube interaction time (%).

Notably, we opted to not attempt to quantify all eligible behavior events to maintain high event detection specificity and a low noise floor at an affordable runtime cost. The primary goal for many studies is accurate comparison of treated animals vs control animals, while the quantification of total events is often of secondary importance. As a result, we opted to bias our quantification to reduce false-positives over fewer false-negatives. For example, for rearing, we quantify rearing events against the three side walls only. For climbing, we only quantify events where the animal reaches the highest part of the food hopper.

### MOT accurately tracks position and health metrics of group-housed animals

To determine the accuracy of MOT-derived health metrics, we established a manually annotated dataset of 13 1-hour long videos for all major behavior types (food and water intake, sleep, climbing and rearing, and aggression) (Figure 3C, Supplementary material). The dataset was gathered from the Allentown Jag75 DOME version and encompasses 2–5 mice per cage, black and white mice, and contains labels for the behavioral state of each mouse in each frame, resulting in a wide variety of conditions. Additionally, we manually labeled a subset of the data for head and body bounding boxes to quantify the accuracy of MOT-inferred bounding boxes and the resulting activity, social distance and location metrics. To determine the accuracy of mouse segmentation and bounding box generation, we compared the human annotated bounding boxes against MOT-inferred bounding boxes, quantifying their average overlap area as well as percent of frames with mis-identified bounding boxes (with less than 75% of overlap to ground truth). Additionally, to estimate the false-positive and false-negative detection rates, we compared the frames annotated by human annotators associated with a behavior (i.e. rearing, climbing, etc.) to MOT-predicted event frames. Frames that were predicted by MOT to contain a behavior but not annotated as such by humans constitute false-positive detections, and frames annotated by humans to contain a behavior but not by MOT constitute false-negative detections (Figure 3C).

The results indicate that the current version (MOT v1.8) achieves a high specificity (low false-positive rate) in tracking and behavior estimation. The average shared area between human and MOT generated bounding boxes is 97.8%, and the average mis-identification rate is less than 0.5%, whereby the errors arise from some extreme occlusion events. Therefore, metrics based on tracking (such as activity, social distance and cage location) are similarly highly accurate. Sleeping, eating, drinking, rearing and climbing behaviors were detected by MOT with high specificity (sleeping: 97.8%, drinking: 98.5%, eating: 82.3%, aggression: 81.2%, rearing: 99%, climbing: 99.9%), indicating that when MOT logs an event, it is usually a true event. Metrics varied in false-negative rates, with sleeping, eating, aggression and drinking averaging about 20%, rearing at 50% false-negatives and climbing at 85% false-negatives, highlighting areas of improvement for future versions of the MOT, particularly for rearing and climbing. Despite the currently relatively high false-negative rates for rearing and climbing (which means that many events are not counted), the metrics are still useful since the relative differences between treated and control groups are accurately captured. Analysis of event distribution between MOT and human annotations largely matched (Figure 3C), indicating that MOT can accurately and specifically identify relative changes in levels of behavior.

### MOT accurately tracks individual group-housed animals

**Figure 4:**
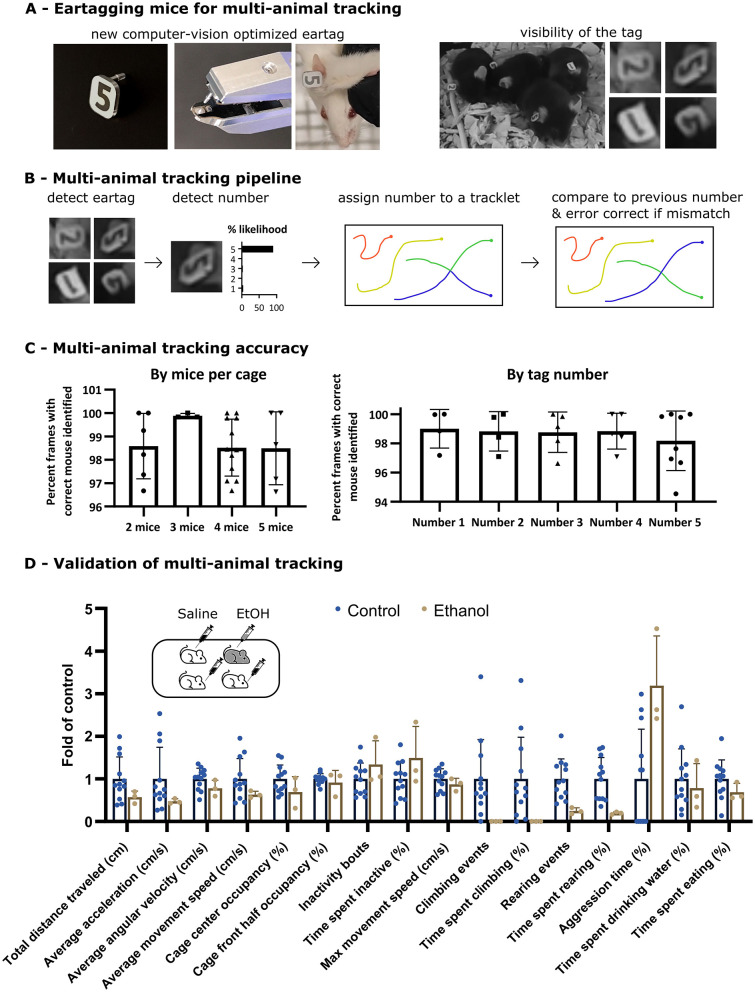
Multi-animal tracking. **(A)** Physical tagging of mice using computer-vision compatible ear tags. Lightweight metal tags with an offset male pin were designed for deep and accurate placement to the topmost portion of the mouse ear. Positioning of the tag directly affects the angle and therefore frequency of tag visibility, which directly affects multi-animal tracking accuracy. **(B)** Two neural networks from the MOT pipeline detect the ear tag and classify the ear tag as a number. While ear tags are not visible every frame, each time an ear tag is detected, it is assigned to a mouse and compared to the current expected mouse ID. In case of mismatch, the mouse tracklet is updated. **(C)** Accuracy of multi-animal tracking. A total of 18 C57BL/6J and 5 BALB/c mice (male and female) at 2–5 mice per cage were ear tagged, recorded and manually labeled for correct identity. Manual ID was compared to MOT-identified ID and the percent of correctly identified frames was calculated per mouse. Results were replicated in a secondary cohort (Supplementary Figure 2). **(D)** To validate accurate individual mouse tracking, 3x cages of 3 month old C57BL/6J female mice were injected either with saline (3× mice) or 20% ethanol (1x) in mixed cages. Additionally, one cage with 3 saline-injected mice was added to the control for a total of *N* = 3 mice for ethanol injected animals and *N* = 12 mice for vehicle injected animals. Data from 3h post-injection was analyzed for metrics, showing a substantial decrease in climbing and rearing events, reduction in speed and increased aggression. These behavioral changes were verified from video recordings after the study. Statistical significance was assessed via multiple unpaired *t*-tests using False Discovery Rate approach to correct for multiple comparisons at FDR Q = 5%. No tests were identified as statistically significant after multiple test correction. Before FDR correction, *p* < 0.05 for Rearing events, Time spent rearing and Aggression time.

While MOT can perform tracking with or without ear tags, occlusions and clumping inevitably lead to loss of unique animal identity over time, unless a method for re-identification of the animal exists. As a result, when mice are tracked by MOT (or any other existing computer vision pipeline) for extended time without markers, they swap identities, which leads to “pseudo-averaging” of the data. This is a substantial barrier for individual animal tracking. As a result, the whole cage must be treated as a single replicate. This reduces the power of the study and can be a fundamental limiting factor if differently treated mice (or mice of different genetic background) are co-housed. To solve this, we initially sought to co-opt an existing ear tag, so we tested all ear tag types on the market for suitability for overhead computer vision tracking. We found that no existing tag was suitable. To be frequently visible, the tag must be placed on the very topmost part of the ear which contains little skin. The tag must also remain positioned upright, be biocompatible, resistant to attempts at chewing and pulling the tag by the animal and their cage mates. Use of existing tags in that area either consistently resulted in the loss of the tag (for pin-based designs), wrong positioning/angulation of the tag (for ring-based designs) or simply lacked robustness or sufficient display size. We therefore engineered our own ear tag and corresponding tagger, finally featuring an edge-displaced male pin, lightweight biocompatible metal base, number generation without coloring or additional material, and a tagger specially designed to enable accurate placement in the narrow top-most part of the ear deep against the base of the ear (Figure 4A). As a result, the tag has high visibility, high stability and high frequency of visibility to the camera, often appearing every 5–10 s during normal activity.

To link this tag to our computer vision pipeline, we added a neural network to MOT that detects the tag when visible, identifies the number on the tag and assigns the number to the associated mouse. If the newly assigned number conflicts with the previously assigned number, the system assumes a previous mixup and automatically applies error correction (Figure 4C). To quantify the effectiveness of the system, we manually annotated animal IDs across a total of 70 min of videos from cages housing 2–5 black and white mice (total number of mice: 23) and compared the results to MOT-identified ID. The analysis revealed that each mouse was identified correctly in over 96% of the frames, with average accuracy of 98% and mis-identification rate ranging from 3.5% to 0%. Similarly, all individual tag numbers (from 1–5) appear to perform effectively, with no clear drop in accuracy for any (Figure 4C). These results were replicated in a secondary cohort of mice across a total of 19 h of video (Supplementary Figure 2). To validate the accuracy of individual tracking in group-housed context, we treated C57BL/6J mice with intra-peritoneal injections of either 1.5g/kg ethanol (20% ethanol in 0.9% saline solution—one animal per cage) or saline solution (the remaining 3 animals per cage), and we tracked their metrics over the subsequent 3 h. While no metrics showed statistically significant differences after FDR correction, the treated mice (replicated across three cages) showed reduced mobility, an almost complete loss of rearing and climbing ability, as well as an increase in being engaged in aggression (Figure 4D). Review of the video footage indicated that increased aggression was initiated by cagemates. However, vehicle-treated mice retained their ability to rear and climb and showed little deviation in activity (Figure 4D). These results are in line with previously published effects of ethanol on mice (Krsiak, 1976) and indicate that the system can accurately track one animal from a group of multiple animals.

### Smart Lids can rapidly identify the health effects of various treatments

**Figure 5:**
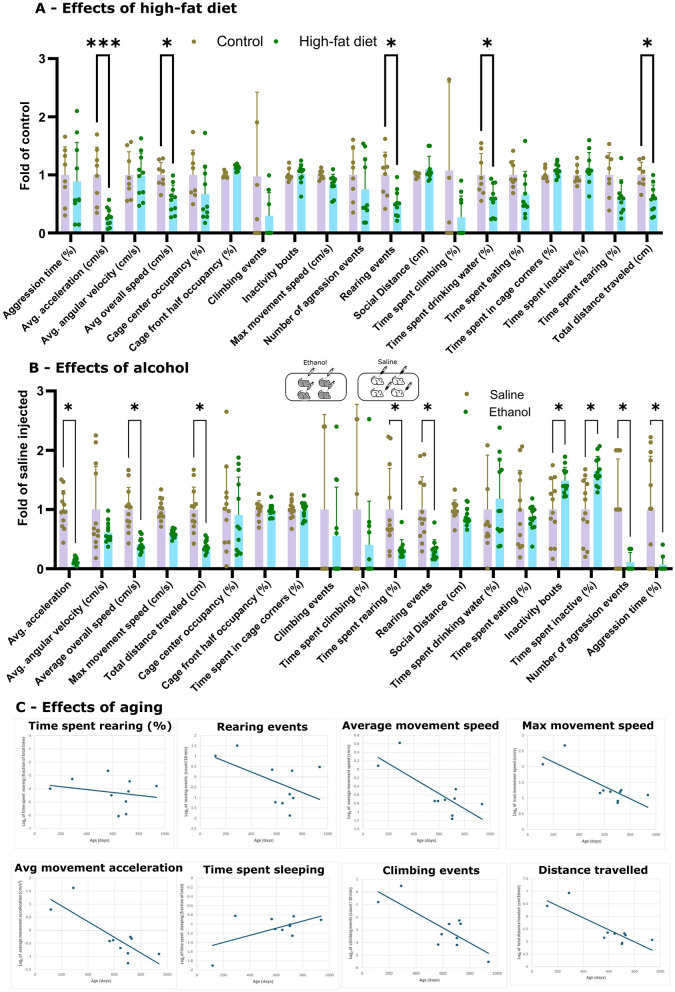
Effects of high-fat diet, alcohol and aging on health and behavior of mice. **(A)** High-fat diet. 3 month old female C57BL/6J mice were fed high-fat diet (45% kcal from fat) or standard chow for 1 month and metrics were recorded for 48 h after habituation for 3 days. Control group: *N* = 8, HFD group *N* = 10, with 3 cages per group. See Supplementary Figures 3, 4 for body weight data and replication data, respectively. **(B)** Ethanol. Ethanol (1.5 g/kg in 0.9% saline) or 0.9% saline vehicle was administered through an intraperitoneal injection to 3 month old C57BL/6J mice, at *N* = 3 cages and total of *N* = 12 mice per group. Unlike in the experiment described in Figure 4, either all mice in the cage received ethanol or all received vehicle; mice were recorded for 4 h post-injection. **(C)** Aging. C57BL/6J mice of different ages were measured for 48 h for 8 metrics. Each dot represents the average of an age group, with a total of *N* = 19 mice used across 10 cages (details in Supplementary Table 1). Statistical analysis **(A, B)**: assessed via multiple unpaired t-tests using False Discovery Rate approach to correct for multiple comparisons at FDR Q = 5%. *p < 0.05, ***p < 0.001 after multiple test correction.

We were interested in testing the effects of two well-known insults to health: high fat diet and old age. In addition, we were interested in whether increased aggression would remain if ethanol treatment was administered to all cagemates. To test the effects of high-fat diet, we fed C57BL/6J mice ad-lib high-fat diet (45% kcal from fat) for 1 month and tested them for 72 h under Smart Lids (Figure 5A). We identified substantial differences in multiple behavior and health parameters. Mice on high-fat diet showed reduced mobility, reduced time spent eating, drinking, climbing and a trend toward increased time spent in the center and front of the cage (Figure 5A). Despite the lack of major obesity, the body weight of the mice fed high-fat diet was significantly higher than that of the mice on standard diet (29 g vs. 21 g, *p* < 0.0001; Supplementary Figure 3). These effects were observed in a replication cohort (Supplementary Figure 4). Additionally, reduced mobility (Han et al., 2021), reduced water intake (Volcko et al., 2020), and altered time spent eating (Davis et al., 2021) have been reported in the literature.

For testing the effect of ethanol, we treated whole cages of 3 month old female C57BL/6J mice either with saline or 1.5 g/kg ethanol injections, administered via intra-peritoneal injections (*N* = 12 mice across *N* = 3 cages per each group). As previously observed, mobility, rearing and climbing reduced while inactivity time and bouts increased. However, on this occasion, aggression flatlined across the treated cages, confirming the hypothesis that the untreated mice increase aggression toward an intoxicated, lower mobility cagemate vs the other way around (Figure 5B).

Finally, to study the effects of aging, we measured a total of 19 wild-type C57BL/6J mice (of both sexes) across 10 cages aged 113–933 days old (Figure 5C). Details of animal ages and sexes are listed in Supplementary Table 1. This study was performed without ear tags as part of an earlier iteration of DOME and assessed 8 metrics. Aging showed clear effects on almost all metrics. Time spent rearing, climbing, and mobility all decreasing with advanced age, while sleep time showed little systematic change. Notably, different metrics exhibited declines that began at different ages and progressed at different rates, suggesting that multiple behavioral dimensions are sensitive to aging, and that aging affects behavior in a dimension-specific manner.

Given these distinct age-related trajectories, we performed an exploratory feasibility analysis to test whether the eight behavioral features could be combined into a single composite estimate of “Digital Bioage”. Inspired by the conceptual framework of the digital frailty index (Ruby et al., 2023), we used a simple regression to map aggregated behavioral features onto chronological age. Unlike Ruby et al. (2023), we chose chronological age as the reference target. The resulting regression captured a clear directional relationship between predicted age and chronological age (Supplementary Figure 5). While preliminary and not intended as a validated biomarker, this feasibility demonstration suggests that multivariate behavioral signatures hold promise for future studies examining biological aging and longevity, and we are actively expanding our dataset to enable development of a generalizable model.

## Conclusions

This work presents a robust and scalable solution for high-content behavioral monitoring in group-housed rodents, addressing long-standing challenges in preclinical research such as low throughput, stress-induced variability, and poor translational fidelity. By combining a low-cost Smart Lid system with a custom-designed computer vision pipeline (MOT) and purpose-built ear tags, we achieve reliable, identity-preserving tracking of multiple unrestrained animals in their home environment, building on advances in automated home-cage monitoring and multi-animal tracking.

The system outputs 21 health-related behavioral metrics, including activity, feeding, drinking, aggression, and sleep, enabling comprehensive phenotyping without human intervention. Validation against manually annotated datasets shows high specificity across most behaviors and identity tracking accuracy exceeding 95%.

Together, these capabilities position the Smart Lid system as a practical, scalable tool for continuous health estimation in both academic and industrial settings. We anticipate that such systems will become critical infrastructure in modern animal research, improving both reproducibility and welfare while unlocking new possibilities for longitudinal and high-resolution studies of behavior and health.

## Data Availability

The datasets presented in this study can be found in online repositories. The names of the repository/repositories and accession number(s) can be found below: https://drive.google.com/drive/folders/1ZJBuFehQpre0XMz3r7P8LIu4VxLoQVFO?usp=sharing.
