# Supplementary material for: Smart Lids for deep multi-animal phenotyping in standard home cages
Source: Front Behav Neurosci. 2026 Jan 20;19:1696654. doi: 10.3389/fnbeh.2025.1696654 (PMC12869192; doi:10.3389/fnbeh.2025.1696654)
Supplement: Supplementary file 1 [file Data_Sheet_1.pdf]

Supplementary Figures

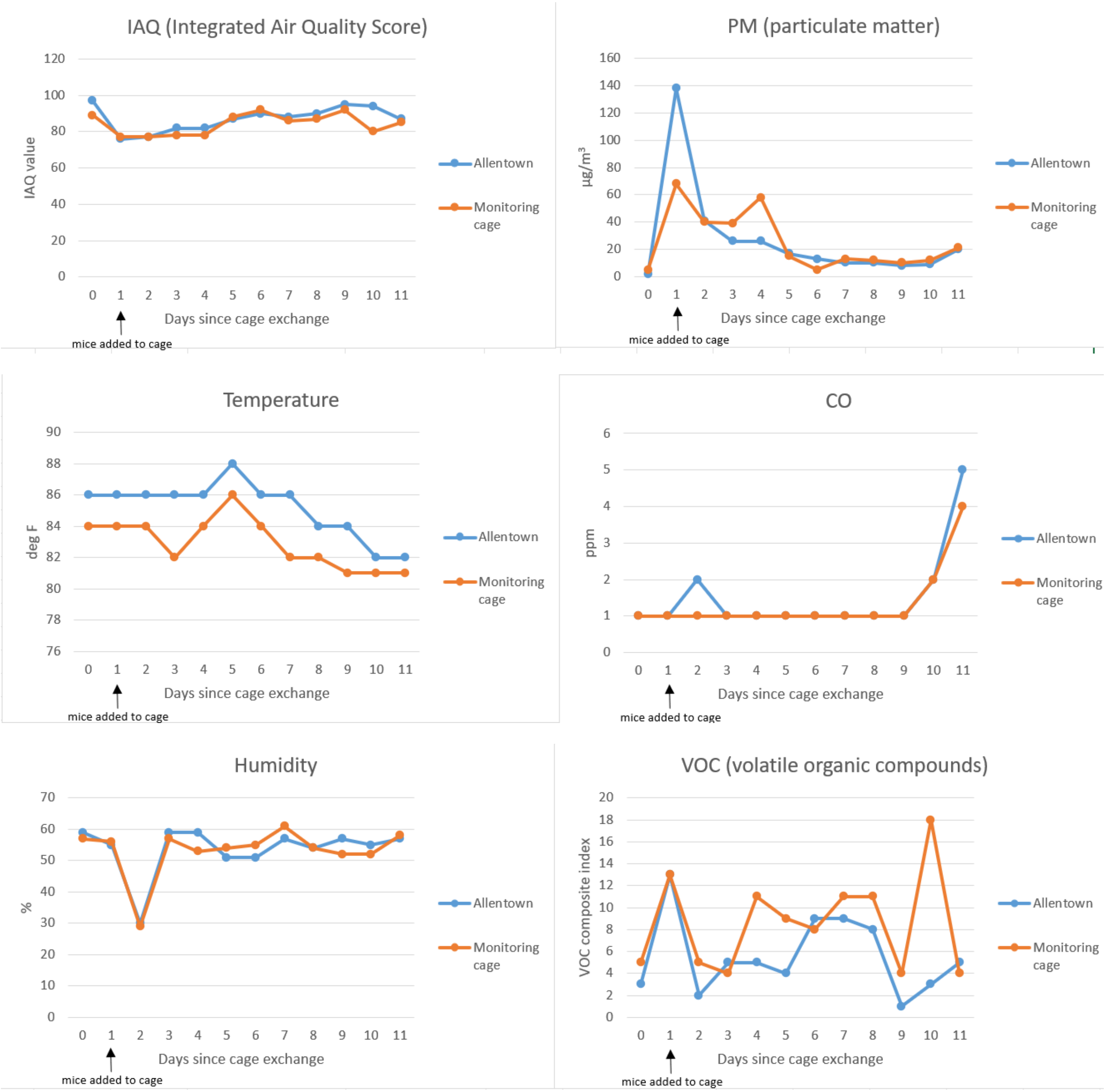

Supplementary Figure 1. Air quality and temperature evaluation for cages with Olden smart lids vs regular lids.

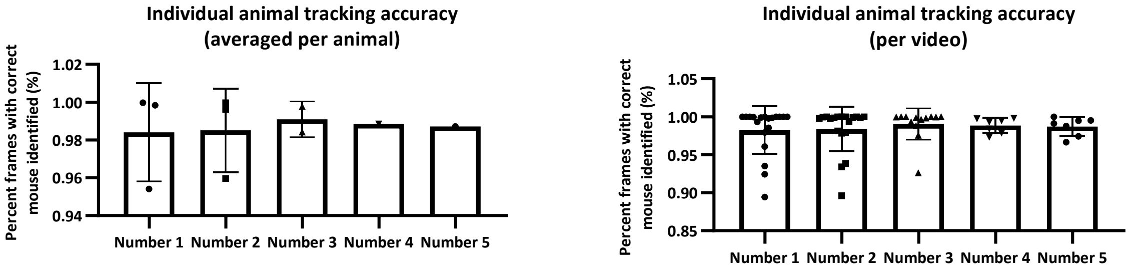

Supplementary Figure 2. Replication of eartag tracking accuracy testing described in Figure 4C. A total of 19 one-hour-long videos from a total of 3 cages of 3 month-old eartagged BALB/c mice containing 2,3 or 5 animals respectively were analyzed as described in Figure 4C.

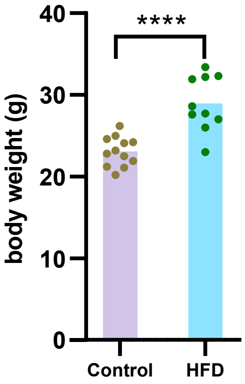

Supplementary Figure 3. Body weights of mice described in Figure 5A. Statistical significance was assessed using a parametric unpaired t-test. ****: $p<0.0001$

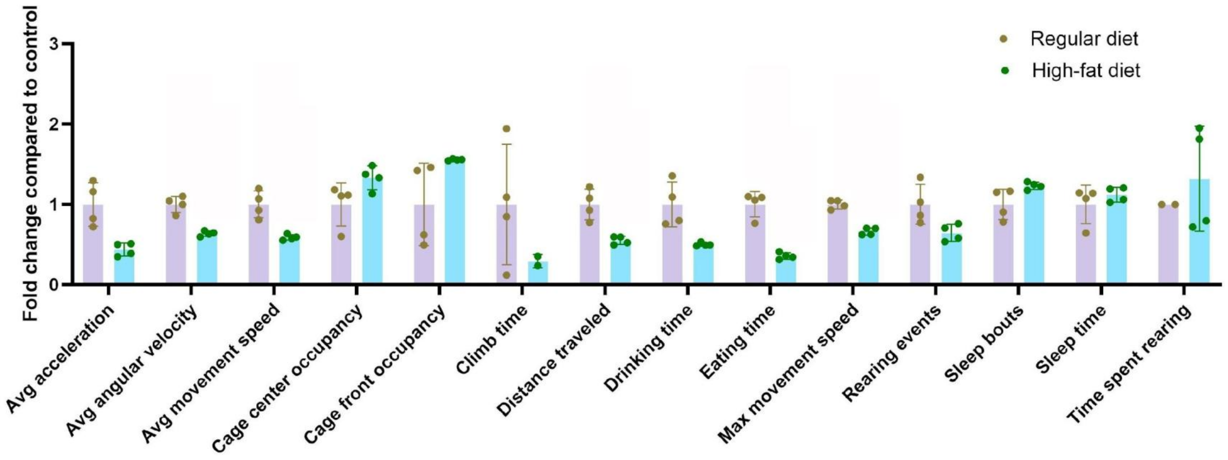

Supplementary Figure 4. Replication of experiment described in Figure 5A.

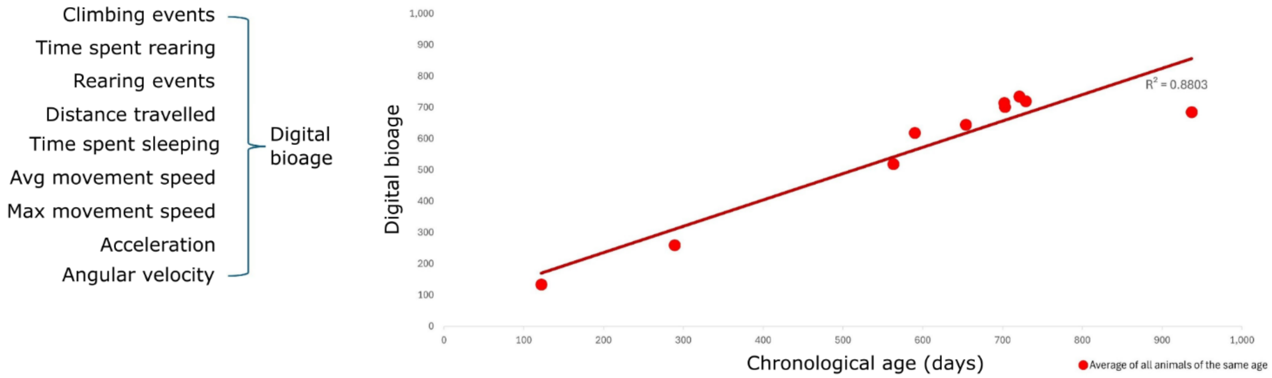

Supplementary Figure 5. Digital Bioage. Eight metrics measured in Figure 5C were trained against chronological age using simple regression to map aggregated behavioral features onto chronological age. A total of N=19 male and female C57BL6/J mice ranging from 113 days old to 933 days old were used (age and sex details in Supplementary Table 1). Each cohort of mice was measured for at least 48 hours. Linear regression was used to generate a set of weights for each metric to provide one compound number that correlates with the chronological age of mice.

Supplementary Methods

Air quality testing experimental setup

One Standard Allentown cage and one modified video monitoring cage were tested side-by-side. Experiment started with a clean new cage and food hopper with clean bedding and new food. Each cage

contained 4x adult female C57BL6/J mice (average ~6 months old). Air quality scores were measured daily. Air quality scores assessed: IAQ, temperature, humidity, PM (particulate matter), CO and VOC (Volatile Organic Compounds – a composite metric of airborne volatile compounds, including ammonia).

Day 0: empty cages. Day 1 -11 mice in the cage (until next cage exchange).

Environmental measurements were collected to confirm that the instrumented lid does not introduce detectable stressors. Airflow, temperature, humidity, and CO_2 were measured at multiple cage locations and compared against standard ventilated lids (Supplementary Fig. 1). No differences were detected. The system does not contain fans or moving parts, and audible sound pressure levels were indistinguishable from background room noise. Visible-light exposure is identical to standard conditions because the lid does not emit visible light and does not block ambient illumination.
Near-infrared illumination is non-visible to mice and does not increase cage temperature. Exact wavelength–intensity specifications are proprietary but fall within typical NIR ranges used in rodent imaging systems. These values are consistent with the absence of thermal or ventilation changes observed in Supplementary Fig. 1.

Health metrics false positive and false negative rate analysis

Thirteen individual 1-h videos were scored by a team of blinded annotators. Each behavior was scored for a start and end frame. Videos were scored by MOT v1.8 for rearing, climbing, eating, drinking and sleeping and by MOT v2.0 for aggression. Aggression analysis was performed after the analysis of other metrics – the reason behind a later version of MOT used. Finally, to find the false positive and false negative rate, the overlap of predicted frames vs ground truth human annotated frames was calculated. False positive rate: percent of total predicted frames in which the event did not occur as estimated by human annotation. False negative rate: percent of total frames annotated to contain a behavior event which was classified negative by MOT.

Because aggression is comparatively rare, a separate set of 13 videos enriched for aggression events were used to estimate the false negative rate, and a randomly extracted set of 130 short MOT-predicted aggression events (10-20sec long) were used to quantify false positive rate. Additionally, event bounts rather than frames were used to calculate false positive and false negative rates, due to the somewhat subjective nature of the beginning and end of an aggression bout. Here, false positive rate was calculated as percent of total MOT-classified events (aggression bouts) which had no overlap with human annotated aggression bouts. False negative rate was the total percentage of aggression bouts as annotated by humans that were not classified by MOT.

Furthermore, because individual videos from different cages would vary in the number of actual aggression bouts substantially (1 aggression in 1 video vs 16 in another), we quantified the false negative and false positive rates per cage, then averaged across all cages to avoid skewing the analysis by cages with a high number of events.

Data and videos are available in Supplementary Information, under "Data" folder.

Manual annotation protocol

All videos in the validation dataset were labeled by trained annotators following a standardized annotation guide specifying definitions, start/stop rules, and exclusion criteria for each behavior (sleeping, eating, drinking, rearing, climbing, aggression). Annotators underwent supervised training using a reference set of labeled clips prior to annotating the validation corpus.

Quality assurance

Each annotator's first several videos were reviewed by a senior annotator for correctness, and ambiguous cases were discussed using consensus rules. A subset (approximately 10%) of the dataset was cross-checked by a second annotator to ensure consistency in behavioral definitions. Because videos were not independently annotated in full by multiple annotators, formal inter-rater reliability statistics such as Cohen's κ cannot be computed retroactively; this limitation is acknowledged.

Dataset limitations

The validation dataset was optimized for estimating false-positive and false-negative rates rather than for full multi-class frame-by-frame confusion matrices. As such, precision, recall, F1, and 95% confidence intervals cannot be reliably computed for all behaviors without re-annotating a balanced dataset. Small sample sizes for rare behaviors (e.g., climbing) further limit confidence interval stability. These limitations are noted in the main text.

Because the validation dataset was annotated for event-level presence/absence rather than for full per-frame multi-class labeling, the dataset does not support construction of full confusion matrices or derivation of F1 scores across all behavioral categories without re-annotation. We therefore report false-positive and false-negative rates directly, which correspond to precision- and recall-informing measures within the structure of the current dataset.

Rearing subcategories

The present pipeline detects wall rears but does not distinguish center or hopper rears. Manual inspection of annotated videos showed no consistent preference for center or hopper rearing across experimental groups, suggesting that omission of these subtypes does not bias relative comparisons. Adding finer-grained rearing categories would require additional neural-network modules for spatial-context inference and represent a future development target.

Digital bioage

The digital bioage regression was performed using behavioral features extracted by the proprietary analysis pipeline. Standardized feature scaling was applied internally as part of this pipeline. Because of the mostly male dataset and exploratory nature of the analysis, no covariates (e.g., sex) were included. Given the small sample size, overfitting remains a potential concern; the model is presented to illustrate the feasibility of deriving age-associated behavioral signatures rather than as a fully operational predictive framework. The implemented regression model relies on proprietary feature-engineering components. Larger datasets and open, fully specified models will be required for a generalizable model.

Number of animals	Age (days)	Sex
2	113	M
5	274	F
1	559	M
2	594	M
1	645	M
1	653	M
2	698	M
2	702	M
1	714	M
1	725	M
1	933	M

Supplementary Table 1: Mice used in aging measurements

Supplementary Discussion

Air quality testing

Overall: no systemic differences between standard Allentown and modified video monitoring cages observed. All air quality metrics remained at or above levels recommended for standard animal housing in the AAALAC Guide for the Care and Use of Laboratory Animals (8th edition).

Minor observations include:

- A sharp peak in airborne particulate matter (PM) was observed in both Allentown and video monitoring cages, likely due to fine particulate matter from refilling food. The peak subsided in both cages after 4 days
- A sharp drop in humidity was observed on day two in both cages, likely due to weather conditions.
- Minor increase in CO levels on day 10 and 11 observed for both Allentown and modified video monitoring cages. CO levels remained very low at <5ppm even on the last day of observation and well within the range of the Guide.
