# Supplementary material for: Smart Lids for deep multi-animal phenotyping in standard home cages
Source: Front Behav Neurosci. 2026 Jan 20;19:1696654. doi: 10.3389/fnbeh.2025.1696654 (PMC12869192; doi:10.3389/fnbeh.2025.1696654)

IMPORTANT NOTES:

1. **GOOD TAG PLACEMENT IS CRITICAL AND DIRECTLY INFLUENCES THE ACCURACY OF MULTI-ANIMAL TRACKING.** If you can't see the tag clearly, neither can the camera.
2. Good placement:
    - Top of the ear, close to the base
    - At least 3mm away from the edge of the ear
    - Clearly visible when looking from above
3. If the tag is placed too close to the edge of the ear, it may eventually be ripped out by the animal. If the tag is too far along the vertical part of the ear, it can't be seen by the camera.
4. Use unique numbers. Any number combination is OK.
5. If a tag is lost, retag the animal on the opposite (left) ear. To do that, use your index finger to pull from the right while scruffing.
6. To improve the tag placement: :
    - Fold the ear open with your free hand before tagging
    - Angle the tagger exactly perpendicular to the head
    - Rest your elbows on the table for stability
7. Both ears can be tagged with the same number to buffer against the loss of a tag.
8. If the male or female holders become loose, replace them with the spare parts provided. TIGHTEN SCREWS CAREFULLY AND ONLY LIGHTLY. They are small and can come loose when overtightened.

OldenTag Guide

Video guide:

oldenlabs.com/knowledgebase/eartaggingvideo

Quick guide:

Step 1. Setup the stand. Then insert the male part of the tag into the tagger. Move the male up and down to test for fit. THERE SHOULD BE VERY LITTLE RESISTANCE. If not, discard the tag or trim its sides.

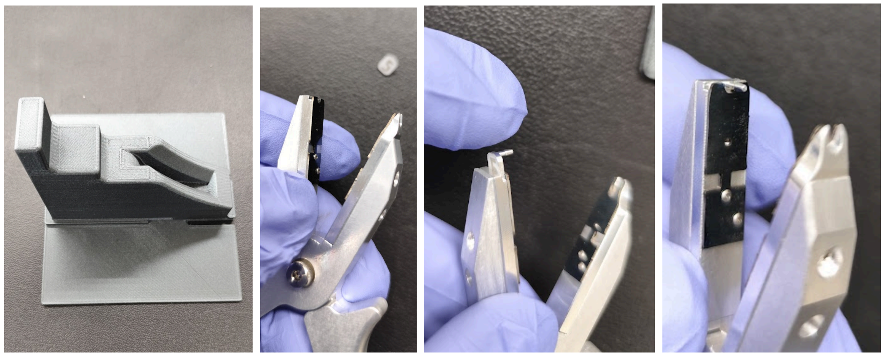

Step 2. Insert the tag female into the tagger. Press down with your thumb, then rotate to release the female. Verify that the female and male are correctly centered by gently tapping the female with the male. DON'T CLAMP FULLY.

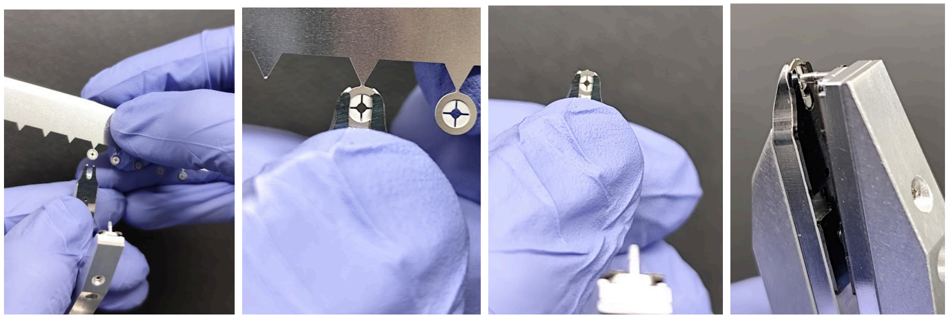

Step 3. Place the loaded tagger onto the stand (male facing down) while you scruff. Double glove your scruffing hand (usually left hand) with the thick black nitrile glove to prevent bite injuries.

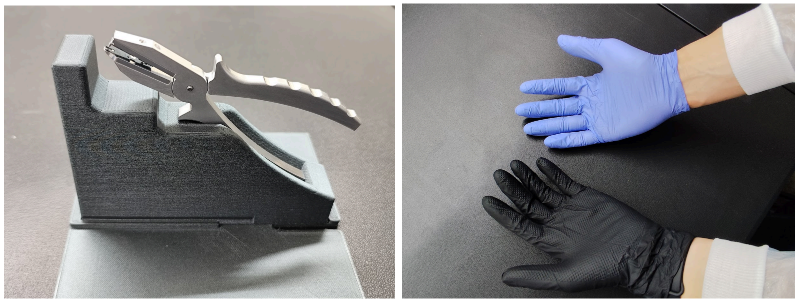

Step 4. Scruff using a modified grip. Pin and hold the animal with your thumb and middle finger. Then use your index finger to pull and immobilize the head from the opposite side.

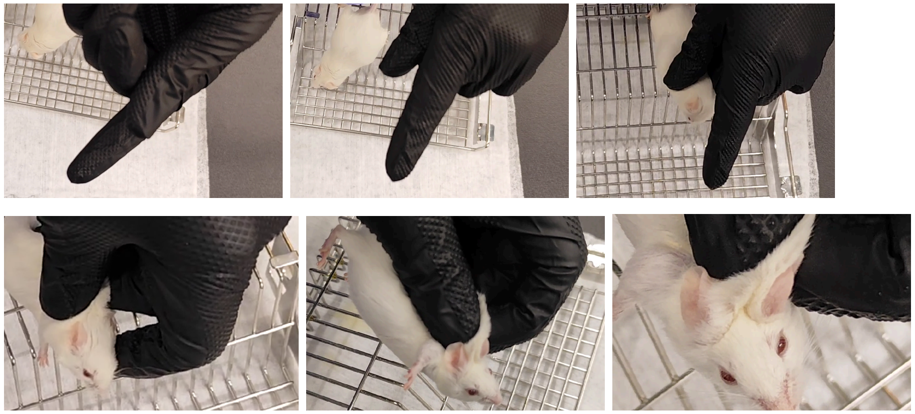

Step 5. With the head stable and immobilized, use your free hand to fold the ear open (mice press their ears against the head). Then bring the tagger to the head at 90 degree angle. Then clamp down at the top base of the ear. **Don't force the tagger open after clamping.**

The tag needs to end up at the top, outside of the ear, be clearly visible when looking from above, and at least 3 mm away from the ear edge to be stable.

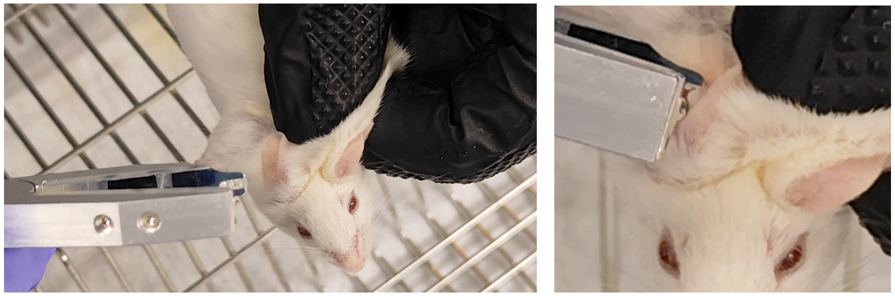

Step 6. To release the tag, lightly release the clamp and gently pull away, perpendicular to the ear. You might feel a small amount of resistance, that is ok.

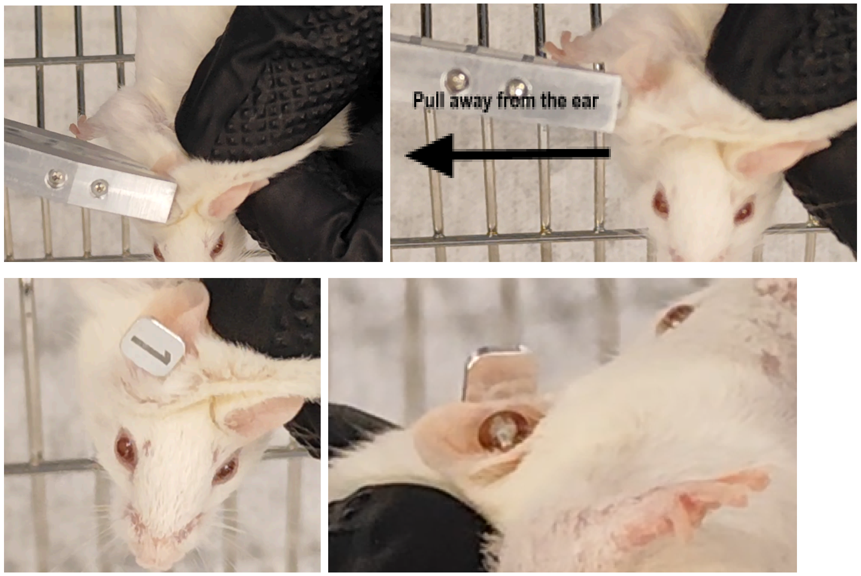

Note: If you feel a lot of resistance, check that hair or skin is not caught in any area and release it with your other hand if so. At worst, place the animal and tagger on the table and use the thin metal sheet from the female holder to push the tag out.

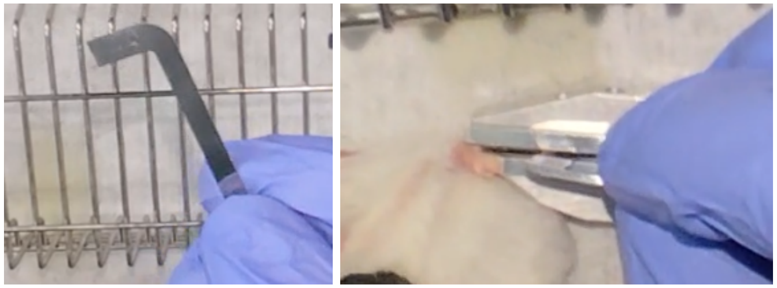

Step 7: To remove the tag (if necessary), wear eye protection, scruff the mouse, insert the cutters underneath the male part and cut the tag. Take care to avoid cutting the ear.

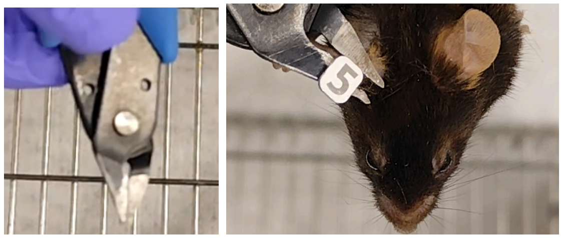

Supplement: Supplementary file 2 [file Data_Sheet_2.pdf]
